# Resveratrol suppresses serum-induced vasculogenic mimicry through impairing the EphA2/twist-VE-cadherin/AKT pathway in human prostate cancer PC-3 cells

**DOI:** 10.1038/s41598-022-24414-z

**Published:** 2022-11-22

**Authors:** Deok-Soo Han, Hyo-Jeong Lee, Eun-Ok Lee

**Affiliations:** 1 Department of Science in Korean Medicine, College of Korean Medicine, Graduate School, Kyung Hee University, 26, Kyungheedae-ro, Dongdaemun-gu, Seoul, 02447 Republic of Korea; 2 Department of Cancer Preventive Material Development, College of Korean Medicine, Graduate School, Kyung Hee University, 26, Kyungheedae-ro, Dongdaemun-gu, Seoul, 02447 Republic of Korea

**Keywords:** Cancer, Drug discovery

## Abstract

Vasculogenic mimicry (VM) is closely related to cancer progression and metastasis, contributing to poor prognosis in patients with cancer. Resveratrol (RES) is well known to possess anti-cancer activity. This study explored the new role of RES in VM incidence in human prostate cancer (PCa) PC-3 cells. The 3-(4,5-dimethylthiazol-2-yl)-2,5-diphenyltetrazolium bromide, transwell invasion, and three-dimensional culture VM tube formation assays were performed to check the cell viability, invasive ability, and vessel-like networks formation, respectively. VM-related proteins were detected by Western blots. The activity of metalloproteinase-2 (MMP-2) was identified by gelatin zymography. Vascular endothelial cadherin (VE-cadherin) mRNA was assessed by reverse transcriptase-polymerase chain reaction. Nuclear twist expression was observed by immunofluorescence assay. RES reduced serum-induced invasion and VM formation. Serum-induced phosphorylation of erythropoiethin-producing hepatoceullular A2 (EphA2) and the expression of VE-cadherin at the protein and mRNA levels were decreased after RES treatment. RES inhibited serum-induced expression and nuclear localization of twist. Serum-activated AKT signaling pathway, including MMP-2 and laminin subunit 5 gamma-2, was impaired by RES. These results suggested that RES may have an anti-VM effect through suppressing the EphA2/twist-VE-cadherin/AKT signaling cascade in PCa PC-3 cells.

## Introduction

Metastasis is the leading cause of death from cancer and occurs through the circulatory system, including the lymphatic system^1^. The blood vessels around cancer supply nutrients and oxygen that play important roles in cancer growth and metastasis^2^. To grow beyond 2–3 mm in diameter, new blood vessel formation from pre-existing ones called “angiogenesis” is necessarily required^3,4^. Since anti-angiogenic drugs target endothelial cells (ECs), it has been proposed that inhibiting angiogenesis can prevent tumor growth and metastasis by destroying blood vessels. However, numerous clinical trials and animal studies have reported that anti-angiogenic therapies have little or no beneficial efficacy, and resistance to these therapies can happen^5–8^. These results indicated that sufficient blood is supplied to the cancer cells through alternative perfusion pathways, even without ECs.

Vasculogenic mimicry (VM) was first reported in 1999 as a unique process by which highly aggressive and metastatic cancer cells generate de novo matrix-rich vascular-like channels in the absence of ECs.^4,9–11^. It effectively mimics the normal blood vessels formed by ECs and is considered as a diagnostic indicator of aggressive and metastatic cancers^12^. In an animal study, anti-angiogenic therapy initially had an inhibitory effect on tumor growth. However, tumor regrowth occurred over a long treatment period. This phenomenon is because tumor cells supplant damaged EC by anti-angiogenic therapy. Moreover, this therapy did not show any effects on tumor growth in VM-competent tumor-bearing mice compared with that in VM-incompetent mice^8^. According to a meta-analysis, the 5-year overall survival (OS) of patients with VM-positive cancer is lower than that of those with VM-negative^13^. VM is closely associated with PCa invasion and metastasis. VM formation has a strong relationship with the Gleason score, lymph node metastasis, and distant metastasis in patients with high-risk PCa. Patients with VM-positive PCa showed lower OS and disease-free survival than those with VM-negative PCa.^14,15^. These reports indicate that the occurrence of VM predicts poor outcomes in patients with cancer. Thus, targeting VM may contribute to overcoming the resistance to anti-angiogenic therapies or may have a synergistic anti-cancer effect by co-administration with anti-angiogenic therapies. Most of all, it would be perfect if drugs had dual effects on targeting VM and angiogenesis.

As naturally occurring compounds, phytochemicals have been studied widely for their beneficial effects, including anti-cancer effects due to their safety. Curcumin, genistein and luteolin have an ability to inhibit VM structure through regulating multiple pathways associated with VM formation^16^. Epigallocatechin-3-gallate (EGCG) in green tea blocks VM process in PC-3 cells^17^. Resveratrol (3,5,4′-trihydroxy-trans-stilbene, RES) is one of the most famous phytochemicals found in red wine, grapes, berries, and peanuts, and is a powerful antioxidant that is helpful in various human diseases, such as cardiovascular diseases and cancer^18^. Although numerous studies have demonstrated that RES has potent anti-cancer properties in various types of cancer^18–20^, only one study has reported that RES suppresses the formation of melanoma VM by inhibiting vascular endothelial growth factor (VEGF) and, VEGF-receptors 1 and 2^21^. RES suppresses proliferation and migration by inhibiting epithelial-mesenchymal transition mediated by TNF-receptor-associated factor 6 in PCa^22^. The anti-metastatic effect of RES has been shown by impairing the AKT/microRNA-21 pathway^23^. Several studies have demonstrated the PCa growth inhibitory effects of RES in in vitro and animal models^24^. In a recent study, it has been announced that serum promotes VM formation in PC-3 cells^25^. Therefore, this study examined whether RES plays a decisive role in inhibiting serum-induced VM in human PCa PC-3 cells, focusing on the EphA2/VE-cadherin/AKT pathway.

## Methods

### Chemicals, antibodies and reagents

**Figure 1:**
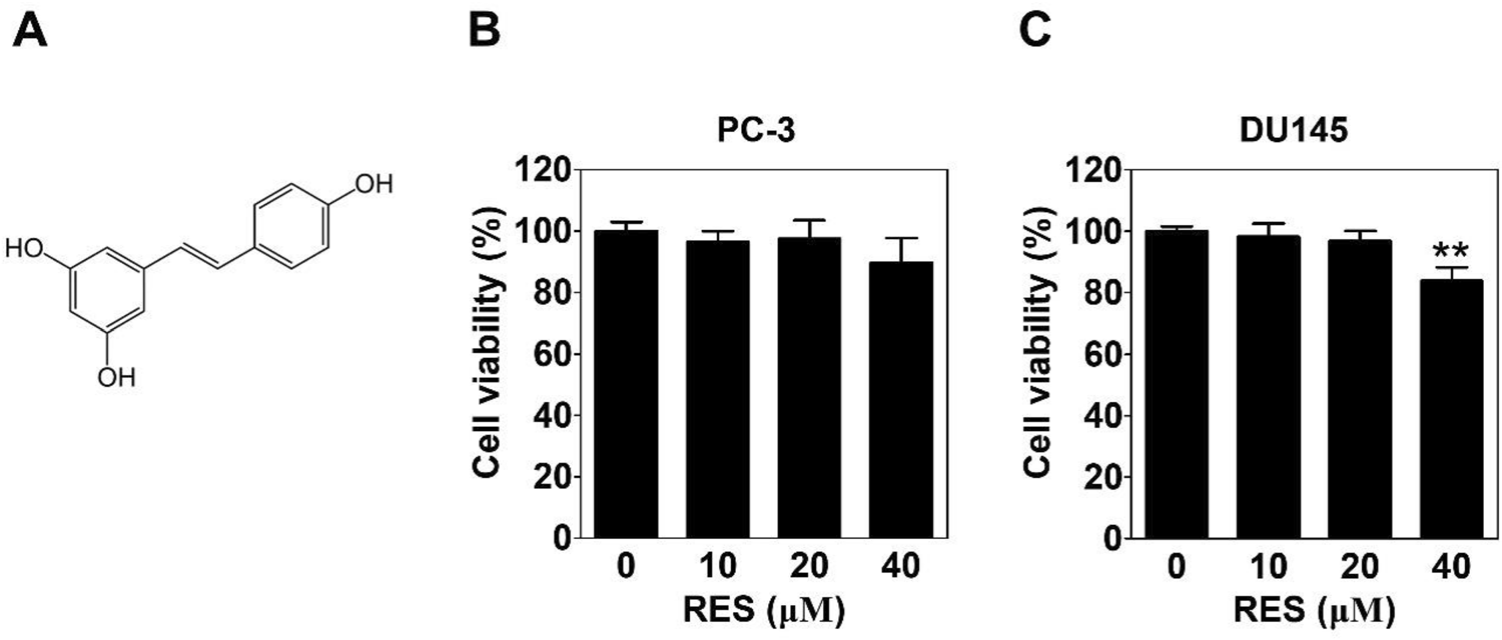
Effect of resveratrol on the cell viability against prostate cancer cells. (**A**) Chemical structure of resveratrol. PC-3 (**B)** or DU145 (**C**) cells were treated with various concentrations of RES for 24 h followed by the MTT assay. Data present as the means ± SD of triplicate determinations.

Resveratrol (RES) (Purity: ≥ 99% as determined by HPLC, Fig. 1A), 3-(4,5-dimethylthiazol-2-yl)-2,5-diphenyltetrazolium bromide (MTT), primary antibody for β-actin and propidium iodide (PI) solution were obtained from Sigma-Aldrich (St Louis, MO, USA). Antibodies specific for p-EphA2 (6347), EphA2 (6997), p-AKT (4060) and AKT (4691) were purchased from Cell Signaling Technology (Beverly, MA, USA), VE-cadherin (AP2724) was obtained from Abgent (San Diego, CA, USA), m-lgGk BP-FITC (sc-516140) was purchased Santa Cruz Biotechnologies (Danvers, MA, USA), LAMC2 (ab96327), MMP-2 (ab86607) and twist (ab50887) were from Abcam (Cambridge, MA, USA). Zymogram-PAG 10% pre-cast gel and developing buffer were purchased from LABISKOMA (Seoul, Korea). All other chemicals were from Sigma-Aldrich.

### Cell culture

Human PCa PC-3 and DU145 cells were purchased from Korean Cell Line Bank (KCLB, Seoul) and were cultured in RPMI 1640 (Welgene, Daegu) with 10% fetal bovine serum (FBS, Welgene, Daegu) and 1% antibiotics (Welgene, Daegu) in a humidified incubator at 37 °C containing 5% CO_2_.

### Cell viability assay

Cells (1 × 10^4^) were seeded on a 96-well plate, and treated with RES (10, 20 and 40 μM) for 24 h in a serum-free culture medium. The MTT assay was performed to determine the cytotoxic effect of RES as described previously^26–28^. Absorbance was measured at 570 nm using a microplate reader (Sunrise RC, TECAN, Mannedorf, Switzerland) and then cell viability was calculated.

### Transwell invasion assay

Cell invasion assay was carried out using a transwell^25^. Costar® Transwell® cell culture inserts (8 μM pore size; Corning Inc., NY) were used after coating with diluted matrigel matrix (1:20 dilution, BD Biosciences, San Jose, CA) to estimate the effects of RES on the invasion of PCa cells. Cells (2 × 10^5^) with RES were seeded on the upper chamber, and the lower chambers were filled with serum for 24 h at 37 °C. Serum-treated cells with or without RES were fixed, stained, and washed, and the cells on the upper chamber were wiped off. The invading cells into the down area were imaged using an inverted light microscope Ts2_PH at 200 × magnification (Nikon, Tokyo, Japan) and counted.

### Three-dimensional (3D) culture VM tube formation assay

VM tube formation was assessed as described previously^25^. A 24-well plate was coated with 100 μl of matrigel at 37 °C for 1 h. Cells (3.2 × 10^5^) were seeded on a matrigel-polymerized plate and treated with serum with or without RES for 16 h at 37 °C. Tubular shapes were imaged using an inverted light microscope Ts2_PH at 40 × magnification and the number of VM structures was counted.

### Western blot analysis

Serum-treated cells (3.2 × 10^5^) on a 6-well plate with or without RES for 24 h at 37 °C were lysed. The protein samples (25–30 μg) from cell lysates were separated by (8–12%) SDS-PAGE at 80 V of constant voltage and transferred onto a membrane (Pall Corporation, Port Washington, NY) for 90 min at 330 mA. After incubation in blocking buffer (5% skim milk or bovine serum albumin [BSA]) for 90 min, the membrane was probed with p-EphA2 (1:1000), EphA2 (1:1000) , VE-cadherin (1:1000), twist (1:200), p-AKT (1:1000), AKT (1:3000), MMP-2 (1:1000), LAMC2 (1:500) and β-actin (1:20,000) antibodies overnight at 4 °C followed by specific secondary antibodies for 2 h at room temperature (RT). Each protein bands were detected using an enhanced chemiluminescence reagent (GE Healthcare, Chicago, IL, USA) and quantified using the ImageJ 1.40 g software (National Institute of Health, Bethesda, MD, USA).

### Gelatin zymography

The conditioned medium (CM) was collected from serum-treated cells with or without RES for 24 h. Equal amounts of CM were separated on Zymogram-PAG 10% pre-cast gel followed by washing with 2.5% triton X-100. And then, the gel was incubated in a developing buffer for 36 h at 37 °C. After staining and destaining, bands were photographed and quantified using the ImageJ 1.40 g software.

### Isolation of RNA and reverse transcriptase-polymerase chain reaction (RT-PCR)

Total RNA extraction was done in serum-treated cells (3.2 × 10^5^) on a 6-well plate with or without RES for 24 h at 37 °C. cDNA synthesis and PCR were then performed. Primers used in this study are listed in Table 1. The PCR products were separated on 2% agarose gel and each PCR product bands were quantified using the ImageJ 1.40 g software.

**Table 1:** Primers used in this study.

mRNA	Primer sequences	Size	Annealing temperature
β-actin	S: GAGAAGATGACCCAGATCATGT AS : ACTCCATGCCCAGGAAGGAAGG	463	60
VE-cadherin	S : GCACCAGTTTGGCCAATATA AS : GGGTTTTTGCATAATAAGCAGG	149	60

### Immunofluorescence assay

Serum-treated cells (1.5 × 10^5^) on an 8-well chamber slide with or without RES for 24 h at 37 °C were fixed with 500 µl of 3.7% formaldehyde and permeabilized with 0.2% Triton-X 100 for 10 min, respectively. The cells were incubated in blocking buffer (5% BSA) for 1 h at RT and probed with twist antibody (1:50) overnight at 4 °C followed by fluorescein isothiocyanate (FITC)-conjugated secondary antibody (1:100) for 1 h at RT. The slide was mounted in 30% glycerol after counterstaining with PI solution. Images were captured using an ECLIPS Ts2-FL microscope (Nikon, Tokyo, Japan) at 400 × magnification.

### Statistical analysis

All results are expressed as the means ± standard deviation (SD) from at least three independent experiments. Student’s *t*-test was performed using the Sigma plot software (Systat Software Inc., San Jose, CA, USA) to determine statistical significance (*p* < 0.05).

## Results

### Effect of resveratrol on the cell viability against prostate cancer cells

To determine the cytotoxicity of RES (Fig. 1A), the MTT assay was performed in RES (10, 20, and 40 μM)-treated PC-3 or DU145 cells. RES treatment at a dose of 40 μM resulted in a slight decrease in the viability of PC-3 cells, which was not statistically significant (Fig. 1B). However, treatment with 40 μM RES significantly decreased in the viability of DU145 cells (Fig. 1C). We conducted subsequent experiments at RES concentrations of 10 and 20 μM.

### Resveratrol suppresses serum-induced invasion and VM tube formation in prostate cancer cells

**Figure 2:**
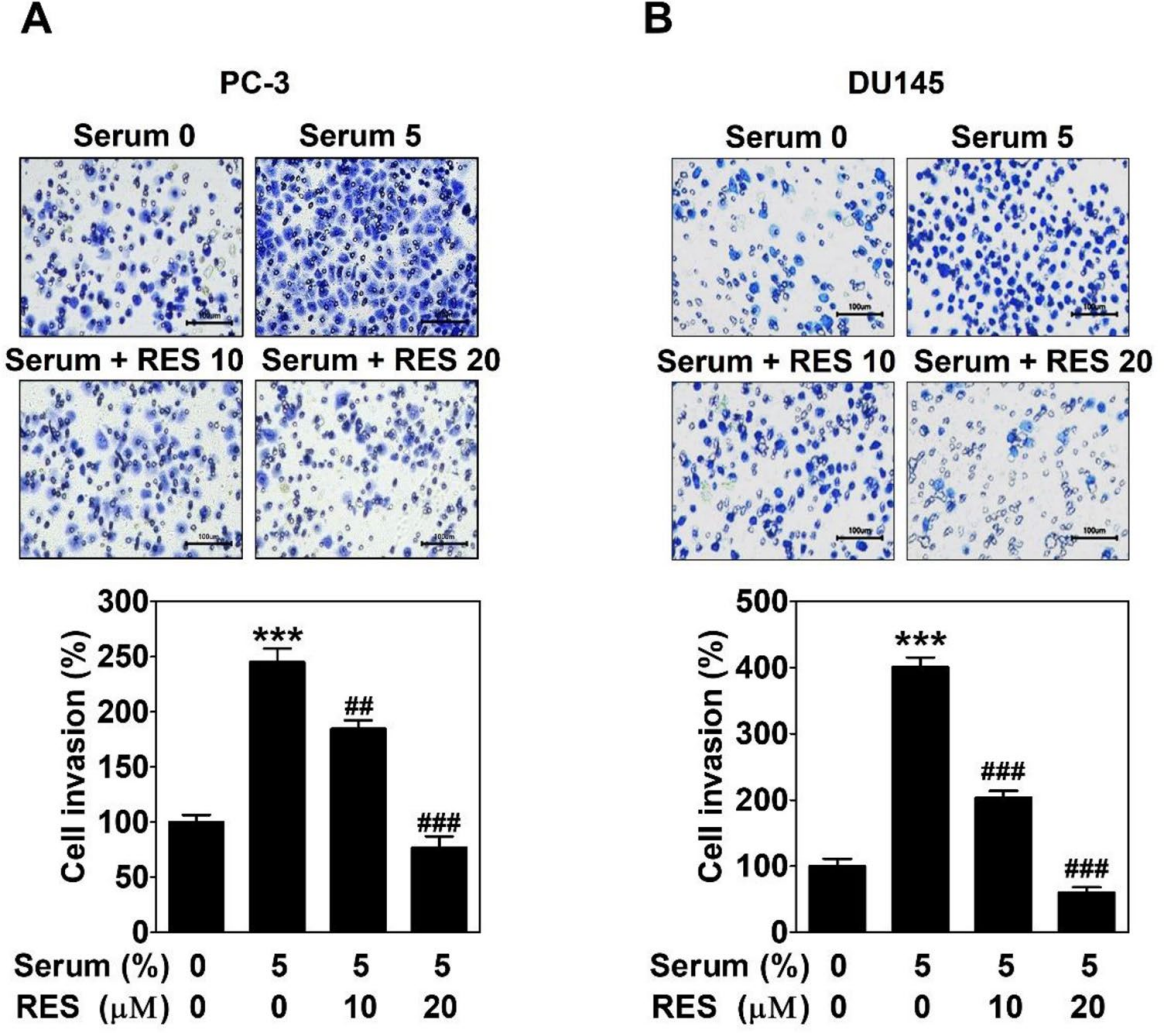
Resveratrol suppresses serum-induced invasion in prostate cancer cells. PC-3 (**A**) or DU145 cells (**B**) with RES seeded on a matrigel-coated up chamber of transwell and serum was filled in a down chamber. After a 24 h-incubation, the cells were fixed and stained. The images were taken with an inverted microscope at 200 × magnification. Scale bar = 100 μm. The number of invading cells was counted and expressed as a percentage of the untreated control group. Data present as the mean ± SD of three independent experiments by analysis of Student’s *t-*test. *** *p* < 0.001 versus untreated control; ##*p* < 0.01 and ###*p* < 0.001 versus serum-treated control.

Transwell invasion assay was carried out to investigate whether RES influences the invasive capacity of PC-3 and DU145 cells. Cells were seeded on a matrigel-coated up-chamber and treated with RES. Serum was added to the down-chamber as a chemoattractant. After 24 h, the induction of cell invasion was significantly increased in response to serum exposure. RES inhibited dose-dependently serum-induced cell invasion in both cell lines (Fig. 2).

**Figure 3:**
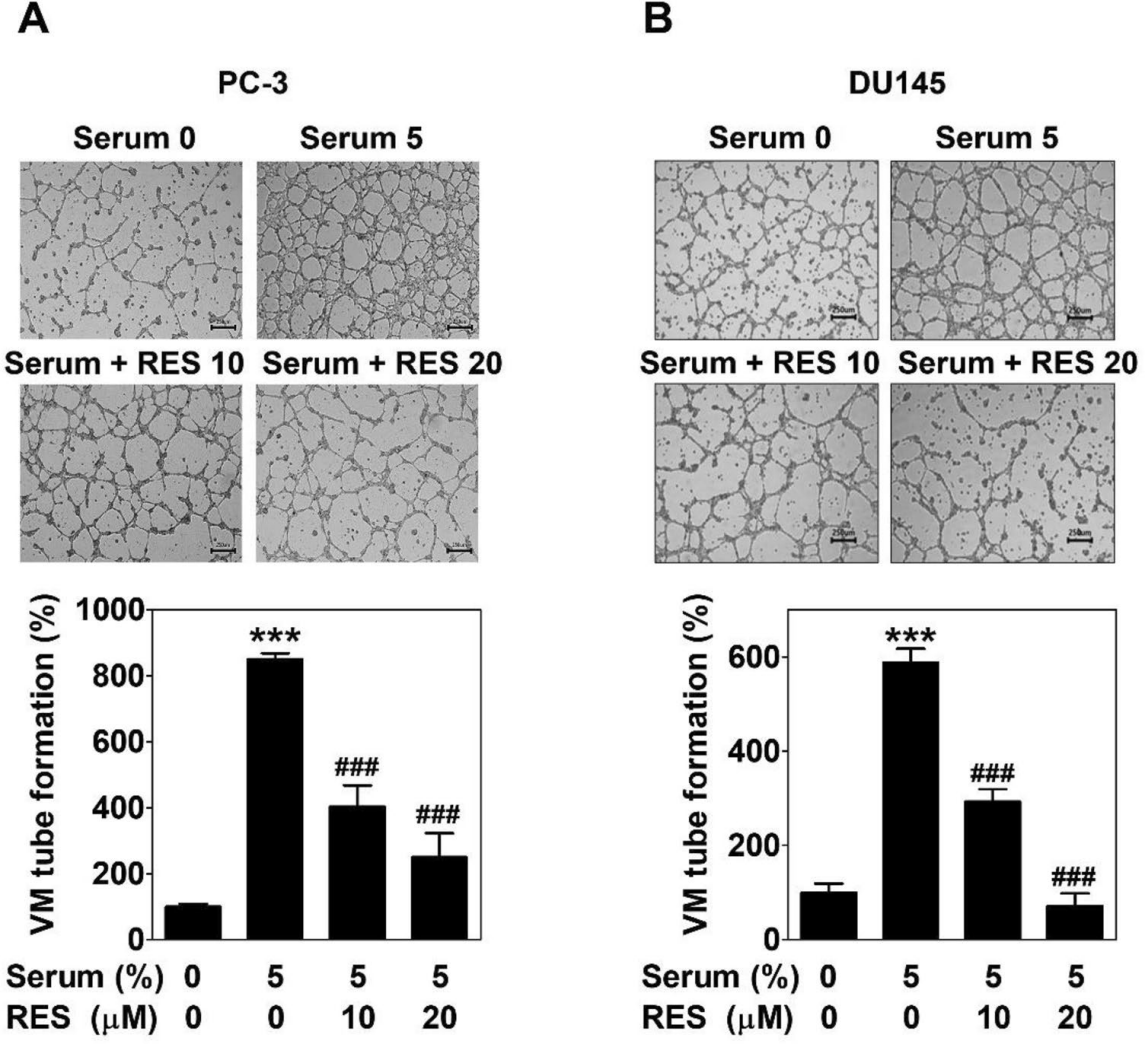
Resveratrol decreases serum-induced VM tube formation in prostate cancer cells. PC-3 (**A**) or DU145 cells (**B**) were seeded on a matrigel-coated a 24-well plate and treated with serum with or without RES. After a 16 h-incubation, the images were taken with an inverted microscope at 40 × magnification. Scale bar = 250 μm. The number of VM tube formation was counted and expressed as a percentage of the untreated control group. Data present as the mean ± SD of three independent experiments by analysis of Student’s *t-*test. ***p < 0.001 versus untreated control; ###*p* < 0.001 versus serum-treated control.

To examine the effect of RES on vessel-like networks formation in PC-3 and DU145 cells, 3D culture VM formation assay was performed on a matrigel-coated plate after treating the cells with serum with or without RES for 16 h. Serum greatly facilitated the formation of perfect tubular shapes. This phenomenon was effectively impeded by RES at 10 and 20 μM in both the cell lines (Fig. 3).

Taken together, these results demonstrate that RES shows both anti-invasive and anti-VM activities in PCa cells.

### Resveratrol inhibits serum-induced the activation of EphA2 in PC-3 cells

**Figure 4:**
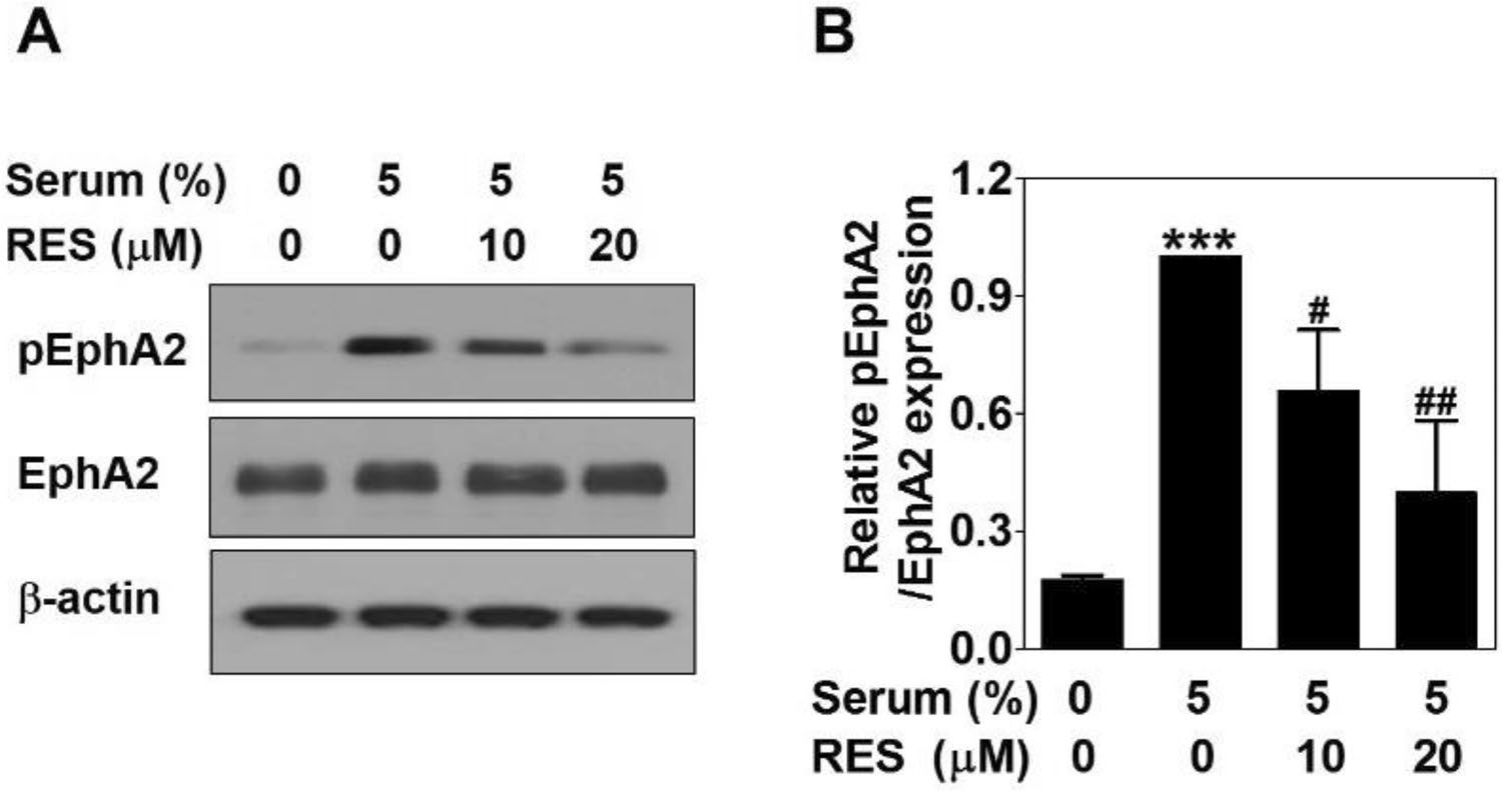
Resveratrol inhibits serum-induced the activation of EphA2 in PC-3 cells. Cells were treated with serum with or without RES for 24 h. (**A**) The same amount of proteins (25–30 μg) were analyzed by Western blot using the phospho-EphA2 and EphA2 antibodies. As a loading control, β-actin was used. (**B**) The bands of proteins were quantified. Data present as the means ± SD of three independent experiments by analysis of Student’s *t*-test. ***p < 0.001 versus untreated control; #*p* < 0.05 and ##*p* < 0.01 versus serum-treated control.

To reveal whether RES affects the activation of erythropoiethin-producing hepatoceullular A2 (EphA2) to suppress serum-induced VM formation, Western blot was conducted in serum-treated PC-3 cells with or without RES for 24 h. The phosphorylation of EphA2 in response to serum was effectively decreased after RES treatment in a dose-dependent manner. However, EphA2 expression levels were not changed by serum or RES (Fig. 4). These results imply that RES causes a marked inhibition of serum-induced activation of EphA2 in PC-3 cells.

### Resveratrol down-regulates serum-induced VE-cadherin expression through decreasing the nuclear twist expression in PC-3 cells

**Figure 5:**
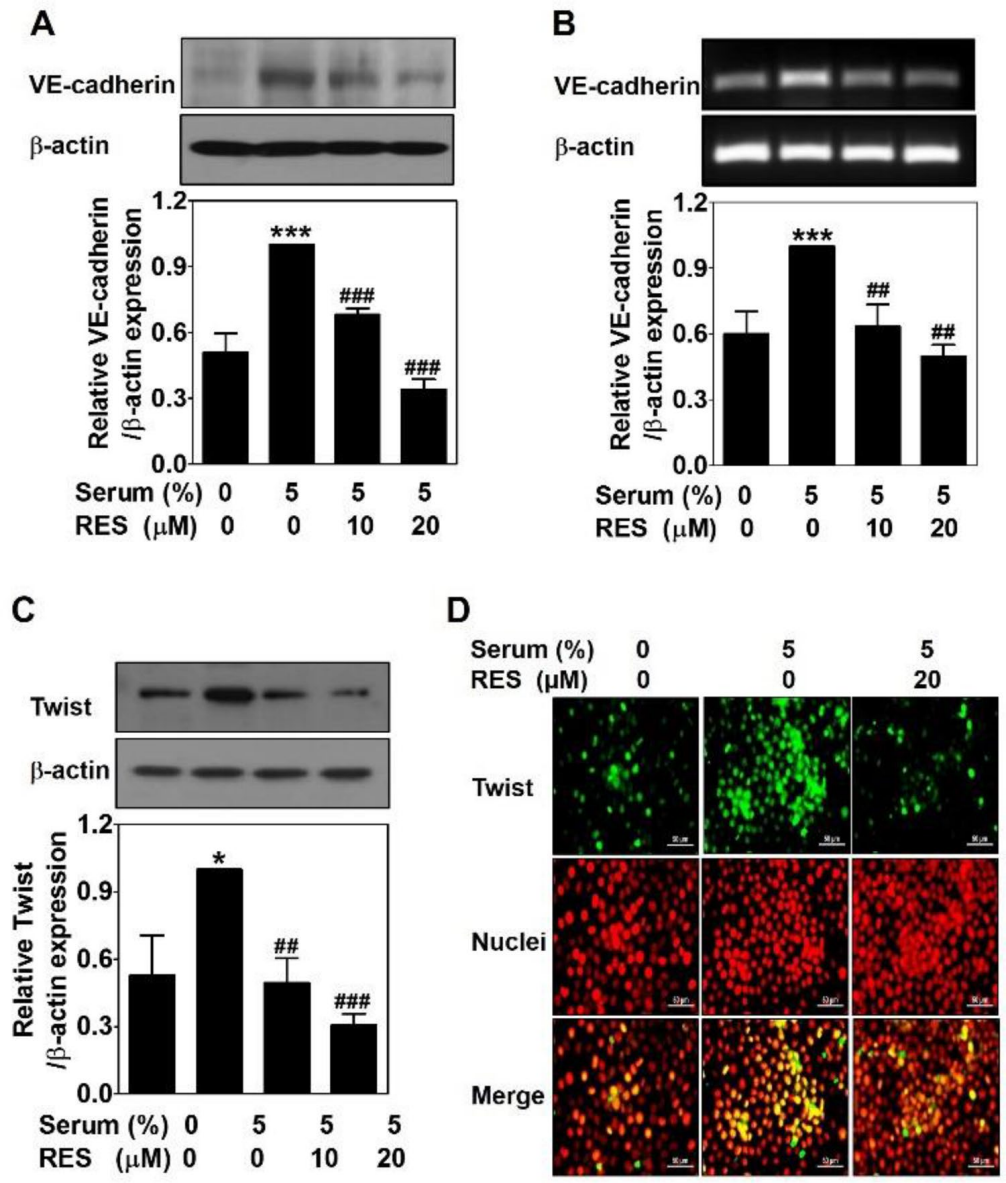
Resveratrol down-regulates serum-induced VE-cadherin expression through decreasing the nuclear twist expression in PC-3 cells. Cells were treated with serum with or without RES for 24 h. (**A**) The same amount of proteins (25–30 μg) were analyzed by Western blot using the VE-cadherin antibody. As a loading control, β-actin was used. (**B**)) The mRNA levels were analyzed by RT-PCR using the VE-cadherin primer. As a loading control, β-actin primer was used. (**C**) The same amount of proteins (25–30 μg) were analyzed by Western blot using the twist antibody. β-actin was used as a control. Data present as the means ± SD of three independent experiments by analysis of Student’s *t-*test. *p < 0.05 and ***p < 0.001 versus untreated control; ##*p* < 0.01 and ###*p* < 0.001 versus serum-treated control. (**D**) The serum-treated cells with or without RES for 24 h were fixed, permeabilized and blocked. After probing with Twist antibody followed by incubation with fluorescein isothiocyanate (FITC)-conjugated secondary antibody, the cells were counterstained with propidium iodide (PI). The images were taken with a fluorescence microscope at 400 × . Scale bar = 50 μm.

To clarify the inhibitory effects of RES on vascular endothelial cadherin (VE-cadherin) protein and mRNA levels were detected by Western blot and RT-PCR in serum-treated PC-3 cells with or without RES for 24 h, respectively. Serum caused an effective upregulation of VE-cadherin protein level, which was significantly downregulated by RES dose-dependently (Fig. 5A). Consistent with the protein expression levels, RES effectively downregulated VE-cadherin mRNA levels induced by serum (Fig. 5B). These results indicate that RES controls VE-cadherin expression at the transcriptional level.

To identify the control mechanism of RES in VE-cadherin expression, twist, a transcription factor of VE-cadherin, was detected by Western blot in serum-treated PC-3 cells with or without RES for 24 h. As expected, serum upregulated twist expression levels, which was drastically reduced by RES treatment (Fig. 5C). To confirm this effect, immunofluorescence analysis was conducted under the same conditions. As shown in Fig. 5D, RES reduced serum-increased twist expression observed in the nucleus.

Taken together, these results indicate that RES downregulates serum-induced twist expression in the nucleus, contributing to a decrease in VE-cadherin expression in PC-3 cells.

### Resveratrol inactivates serum-induced the AKT signaling pathway in PC-3 cells

**Figure 6:**
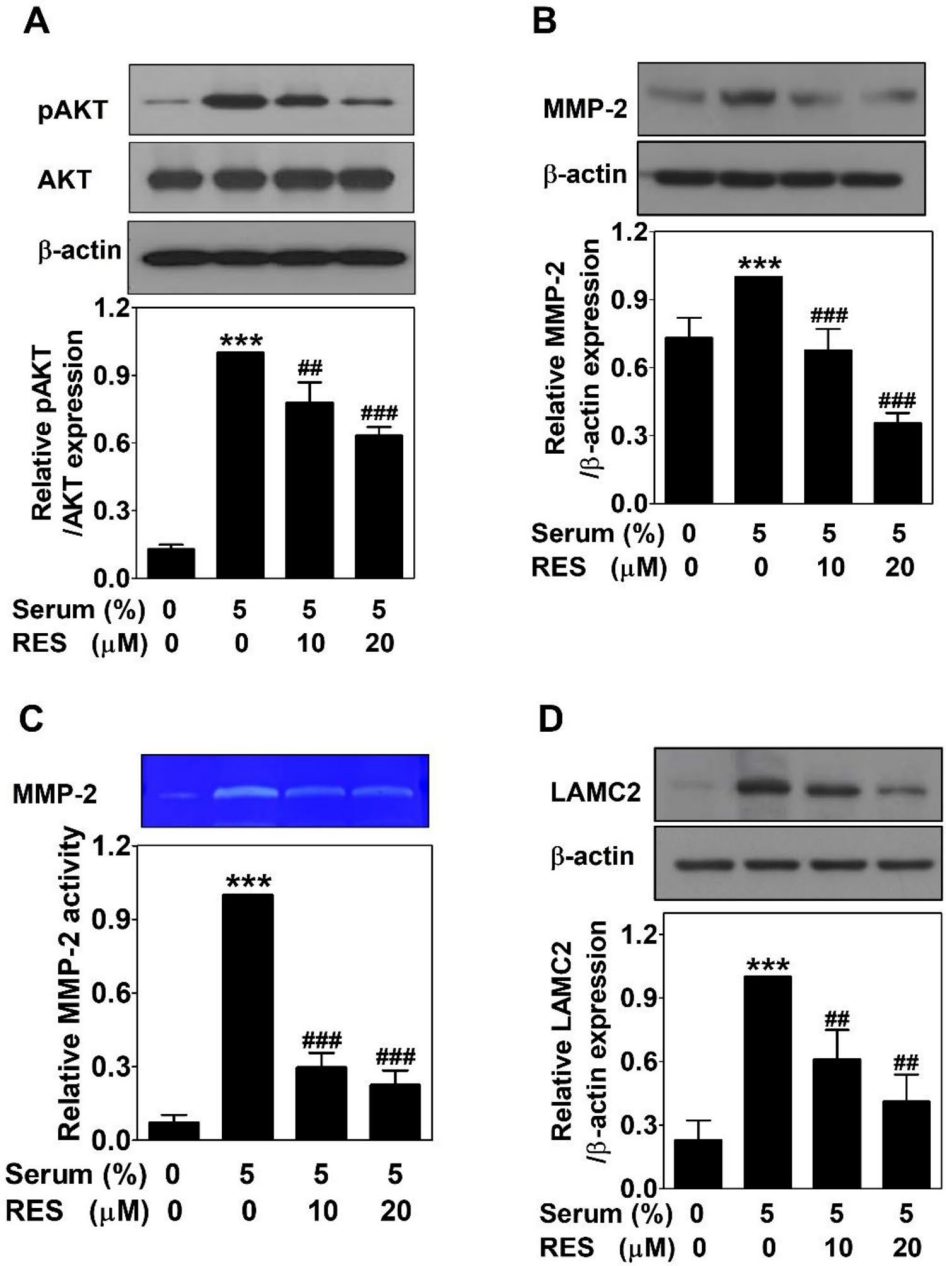
Resveratrol inactivates serum-induced the AKT signaling pathway in PC-3 cells. Cells were treated with serum with or without RES for 24 h. The same amount of proteins (25–30 μg) were analyzed by Western blot using the phospho-AKT, AKT (**A**), MMP-2 (**B**) and LAMC2 (**D**) antibodies. As a loading control, β-actin was used. (**C**) Gelatin zymography was performed using the CM. Data present as the means ± SD of three independent experiments by analysis of Student’s *t-*test. ***p < 0.001 versus untreated control; ##*p* < 0.01 and ###*p* < 0.001 versus serum-treated control.

The AKT pathway was explored by Western blots in serum-treated PC-3 cells with or without RES for 24 h to assess whether this pathway is associated with the anti-VM effect of RES. Serum increased the phosphorylation of AKT but not the expression of AKT. RES treatment blocked dose-dependently the phosphorylation of AKT by serum without affecting AKT expression (Fig. 6A). The expression of matrix metalloproteinase-2 (MMP-2) was upregulated by serum. This effect also inhibited by RES treatment dose-dependently (Fig. 6B). In addition, to assess the activity of MMP-2, gelatin zymography was performed using CM from serum-treated PC-3 cells with or without RES for 24 h. RES effectively impaired the serum-induced activity of MMP-2 (Fig. 6C). Serum-upregulated laminin subunit 5 gamma-2 (LAMC2) was also inhibited by RES treatment dose-dependently (Fig. 6D). These results suggeste that RES suppresses serum-induced VM formation by inactivating the AKT/MMP-2/LAMC2 signaling pathway in PC-3 cells.

## Discussion

As a powerful antioxidant, RES has anti-cancer effects by inhibiting angiogenesis and metastasis and inducing apoptosis in various types of cancer cells^18–20^. However, there is insufficient evidence of a link between RES and blocking of VM formation. In a recent experiment, we demonstrated the following results: (1) serum activates EphA2 and (2) upregulates VE-cadherin expression through increasing nuclear twist expression, (3) which in turn activates the AKT/MMP-2/LAMC2 pathway, (4) contributing to the formation of VM in PCa PC-3 cells^25^. Based on these findings, we explored whether and how RES affects serum-induced VM in PC-3 cells.

As an alternative perfusion pathway, VM is the formation of matrix-rich blood vessel-like shapes by aggressive and metastatic cancer cells^9,10^. It can easily be identified in PCa cells, such as PC-3 cells^15,29^. There is a strong relationship between VM and cancer cell motility^30^. RES suppresses the motility, such as the migration and invasion of PCa^31,32^. As expected, RES inhibited the invasion of serum-treated PCa cells (Fig. 2). In addition, RES blocked complete tubular channels induced by serum (Fig. 3). All the effects were observed at non-cytotoxic concentrations (Fig. 1B and 1C). These results show that RES plays a novel role in inhibiting VM formation in PCa.

EphA2 is a tyrosine kinase-containing transmembrane glycoprotein receptor. A high expression of EphA2 increases the invasion of PCa cells^33^. EphA2 has been considered as a key driver of VM process in various types of cancers, including PCa^34^. In in vitro and in vivo models, EphA2 contributes to tumor growth and VM formation^35^. Its expression and phosphorylation levels are closely related to VM formation^15,34^. Thus, EphA2 is an attractive biomarker for targeting VM. The anti-VM effect of microRNA-141 results from downregulation of EphA2 expression^36^. In this study, the activation of EphA2 by phosphorylation in serum-treated cells was effectively reduced without affecting EphA2 expression after RES treatment (Fig. 4). This result indicate that RES can block VM by controlling EphA2.

The activity of EphA2 is controlled by VE-cadherin that is a main adhesion receptor exclusively expressed in endothelium^10,30,37^. It contributes to blood vessel formation and maintains and controls ECs contacts and vascular permeability. However, aberrant overexpression of VE-cadherin has been observed in highly aggressive and metastatic cancer cells^38–40^. VE-cadherin is one of the first molecules involved in VM signaling pathway. In the absence of VE-cadherin, VM formation does not occur^10,40^. Genistein^41^ and ginsenoside Rg3^42^ inhibit VM formation by downregulating VE-cadherin expression. RES inhibited serum-upregulated VE-cadherin at both the protein (Fig. 5A) and mRNA (Fig. 5B) levels, indicating that RES controls VE-cadherin expression at the transcriptional level. Twist is well known as a transcription factor of VE-cadherin^43,44^. It promotes tumor progression by regulating cancer cell growth, metastasis, differentiation, and angiogenesis^45^. Previous studies have shown that twist can induce VM formation by regulating VE-cadherin expression levels^43,46^. The anti-VM effects of EGCG are mediated by inhibiting twist and VE-cadherin expressions^17^. Polyphyllin I inhibits twist binding to the VE-cadherin promotor, leading to anti-VM activity^47^. RES suppressed serum-upregulated twist expression in the nucleus (Fig. 5D). Taken together, the VM blocking effect of RES is related to the downregulation of VE-cadherin expression by inhibiting nuclear twist expression.

EphA2 co-localizes with VE-cadherin at sites with cell-to-cell junctions. This interaction results in the activation of the phosphoinositide 3-kinase (PI3K)/AKT pathway that is important in regulating cancer progression, such as survival, proliferation, angiogenesis and metastasis^10,30,37,48^. The PI3K/AKT pathway also participates in extracellular matrix remodeling and VM process through activating MMP-14 and -2, facilitating the cleavage of LAMC2, thereby promoting VM-related motility of cancer cells^10,30,37^. EGCG shows anti-VM activities in PC-3 cells through inhibiting the AKT signaling^17^. Also, phytochemicals, such as curcumin, honokiol, and norcantharidin have anti-VM effects through suppressing the AKT pathway^16^. In this study, serum-activated AKT levels were significantly reduced by RES treatment (Fig. 6A). Highly invasive and aggressive cancer cells overexpress MMP-14 and -2 and LAMC2, which help to form a vascular structure lined by cancer cells^49^. The AKT/MMP-2/9 pathway is required for the regulation of VM formation^50^. Serum upregulated the expression of MMP-2 and LAMC2, which was effectively decreased after treating with RES (Fig. 6B and 6D). In addition, the activity of MMP-2 by serum was effectively impaired by RES (Fig. 6C). Taken together, these results verify that RES effectively suppresses the AKT/MMP-2/LAMC2 cascades, contributing to the anti-VM activity of RES.

## Conclusion

This study demonstrates a new role for RES in anti-cancer effects. RES suppressed VM structure formation in PCa PC-3 cells at non-cytotoxic concentrations. This effect is mediated by inactivating EphA2 and reducing twist-mediated VE-cadherin expression, which in turn inactivates the AKT/MMP-2/LAMC2 signaling pathway. This study provides new insights into the functions of RES. However, further work, including in vivo studies, is required.

## Data Availability

The data was available from corresponding author upon reasonable request.

## Abbreviations

BSA: Bovine serum albumin
ECs: Endothelial cells
EGCG: Epigallocatechin-3-gallate
EphA2: Erythropoiethin-producing hepatoceullular A2
FITC: Fluorescein isothiocyanate
LAMC2: Laminin subunit 5 gamma-2
MMP-2: Matrix metalloproteinase-2
MTT: 3-(4,5-Dimethylthiazol-2-yl)-2,5-diphenyltetrazolium bromide
OS: Overall survival
PCa: Prostate cancer
PI: Propidium iodide
PI3K: Phosphoinositide 3-kinase
RES: Resveratrol
RT: Room temperature
RT-PCR: Reverse transcriptase-polymerase chain reaction
VE-cadherin: Vascular endothelial cadherin
VEGF: Vascular endothelial growth factor
VM: Vasculogenic mimicry
